# Bell’s Palsy—Retroauricular Pain Threshold

**DOI:** 10.3390/medicina57030263

**Published:** 2021-03-13

**Authors:** Aleksandar Kopitović, Filip Katanić, Sandro Kalember, Svetlana Simić, Nina Vico, Slobodan Sekulić

**Affiliations:** 1 Faculty of Medicine, University of Novi Sad, 3 Hajduk Veljkova Street, 21000 Novi Sad, Serbia; aleksandar.kopitovic@mf.uns.ac.rs (A.K.); sandrokalember@yahoo.com (S.K.); svetlana.simic@mf.uns.ac.rs (S.S.); nina.vico@gmail.com (N.V.); slobodan.sekulic@mf.uns.ac.rs (S.S.); 2 Department of Neurology, Clinical Center of Vojvodina, 1-9 Hajduk Veljkova Street, 21000 Novi Sad, Serbia

**Keywords:** retroauricular pain, digital pressure algometer, pain threshold, idiopathic Bell’s palsy, mirror-image pain threshold, ephaptic coupling

## Abstract

*Background and objectives:* Non-motor symptoms in the form of increased sensitivity are often associated with the onset of idiopathic Bell’s palsy (IBP). The aims were to determine whether the pain threshold in the retroauricular regions (RAR) in IBP patients and the time of its occurrence is related to IBP severity. *Materials and Methods:* The study was conducted among 220 respondents (142 IBP patients, 78 healthy subjects (HS)). The degree of IBP was graded using the House–Brackmann and Sunnybrook Grading Scales (II—mild dysfunction, VI—total paralysis), whereas the pain thresholds were measured using the digital pressure algometer. *Results:* We found no difference in the degree of the pain threshold between the right and left RAR in the HS group. IBP patients belonging to groups II, III, IV, and V had lower pain thresholds in both RARs than HS and IBP patients belonging to group VI. There was no difference in the degree of pain threshold in RAR between the affected and unaffected side in IBP patients. The incidence of retroauricular pain that precedes paralysis and ceases after its occurrence in groups II and III of IBP patients is noticeably lower and the incidence of retroauricular pain that occurred only after the onset of paralysis is more frequent. Also, we found that the incidence of retroauricular pain that precedes paralysis and ceases after its occurrence in groups V and VI of IBP patients was more frequent. *Conclusions:* The degree of pain threshold lowering in RAR (bilaterally) is inversely related to the severity of IBP. We suggest that the occurrence of retroauricular pain before the onset of facial weakness is associated with higher severity of IBP while the occurrence after the onset is associated with lower severity of IBP.

## 1. Introduction

The facial nerve (FN) provides innervation to the mimic muscles of the ipsilateral half of the face, the posterior belly of the digastric muscle, stapedius, and stylohyoid muscles. It is mainly a motor nerve, but it also contains sensory and parasympathetic fibers that constitute the intermediate nerve which provides taste sensation from the anterior two-thirds of the tongue; enables secretory and vasomotor fibers to reach the lacrimal, submandibular, and sublingual salivatory glands, as well as transmits cutaneous sensory impulses from the external auditory meatus and retroauricular region (RAR) [1,2].

Both inflammatory edema in the FN and a vasospasm of the stylomastoid artery are shown to decrease blood supply [3,4] and result in a neurovascular compression within the narrow canal in the temporal bone, preventing the transmission of nerve impulses from the central nervous system (CNS) and thus resulting in weakness on the affected side of the face. The most common diagnosis of FN paralysis is idiopathic Bell’s palsy (IBP), which accounts for more than 70% of cases and occurs among the general population with an incidence rate ranging from 10 to 53.3 per 100,000 patients across different regions [5,6,7]. Although generally unilateral, it is described that in some rare cases it affects both FNs [1]. The etiology is not fully understood, but viral infections, vascular ischemia, or autoimmune diseases have been postulated to be the most likely pathogenic mechanisms involved [5,8,9].

IBP has different path stages including acute stage (1–7 days), resting stage (8–20 days) and restoration stage (21–90 days) [7]. Although it is a common outpatient problem, for which the diagnosis is straightforward, a number of diagnostic pitfalls can occur, and a long-term differential diagnosis exists [10]. In addition to the weakness of the muscles, dry eyes or mouth, disturbance or complete loss of taste, hyperacusis, and sagging of the eyelids or around the corners of the mouth also appear [6,11]. The most alarming symptom of IBP is the paresis itself but the retroauricular pain (RAP), which is experienced by more than half of patients, has also aroused a great deal of interest. The pain is localized deep in the mastoid region that generally lasts for up to one or several weeks and requires analgesia [1,12].

The pathogenesis of RAP in these patients is not entirely clear [1]. With regard to the fact that while some studies suggest worse outcomes for patients with IBP [12,13,14], on the other hand, other studies have yielded different results [15,16,17,18], entailing the need for defining clearly and precisely whether it is a positive or a negative prognostic factor. RAP occurs in over 50% of patients, of whom one half had it before the onset of paralysis while the second half had it only after the onset of paralysis due to negligence of pre-existing paralysis [1,12,15]. On the account of the presence of the phenomena of central sensitization and areas of referred pain [19], as well as the fact that some studies found that nerve damage can affect the opposite side of the body [20,21], the pain threshold was also examined in the RAR of the opposite side.

The aims were to:

1. determine whether the pain threshold in the RARs in patients with IBP is related to IBP severity;
2. determine whether the time of RAP occurrence is prognostic for IBP severity.

## 2. Materials and Methods

### 2.1. Study Design and Population

This study was conducted at the Clinic for Neurology, Clinical Center of Vojvodina, in the period from October 2018 to June 2020. The study was conducted in accordance with the Declaration of Helsinki, and the protocol was approved by the Ethics Committee of Clinical Center of Vojvodina (the protocol number: 00–20/194; the data of approval: 9th February 2018).

All the patients affected by facial paralysis that reached the neurology department of our hospital have been evaluated. After a detailed neurological examination, we exclude central nervous system involvement. Magnetic resonance imaging of the brain and/or magnetic resonance angiography were performed when clinical history or clinical signs suggested a secondary facial palsy.

The patients included in this study group met the following criteria:

### 2.2. Inclusion Criteria

- patients with unilateral IBP (International Classification of Diseases (ICD), 10th Revision);
- acute phase of IBP (within 7 days from onset) [7], without detectable cause;
- no previous pharmacological therapy for the episode of facial palsy;
- adults between 20 and 65 years of age.

### 2.3. Exclusion Criteria

- facial paralysis due to other causes (Guillain-Barré syndrome, Lyme disease, Ramsay Hunt syndrome, sarcoidosis, cholesteatoma, parotid tumor, traumatic paralysis, iatrogenic…) [22];
- otalgia or RAP due to other causes (trauma, diabetes mellitus, otitis externa or otitis media, mastoiditis, sinusitis, salivary gland disorders, tumors or infected cysts, dental causes, idiopathic…) [23];
- patients presenting with multiple cranial neuropathies, facial contractures, synkinesis, or spasms due to various causes.

Respondents included in the control group were healthy volunteers who met the following criteria:

- never had Bell’s palsy type of motor weakness, nor any form of peripheral or central facial motor weakness;
- never had multiple cranial neuropathies, otalgia, RAP, facial contractures, synkinesis, or spasms due to various causes;
- do not have the presence of any neurological pain syndrome or painful condition;
- normal neurological assessment;
- do not take any analgetic therapy;
- adults between 20 and 65 years of age.

Included in the study were 220 respondents (78 healthy subjects (HS)—mean age 39 ± 19 years and 142 patients with Bell’s palsy—mean age 41 ± 21 years) who met the previously listed criteria.

Based on the anamnestic data and the presence of RAP, in patients with RAP experience, it was determined whether it was a prodromal symptom or it has occurred after the onset of paralysis.

The degree of IBP was determined using the House–Brackmann (HB) and Sunnybrook (SB) Grading Scales. There are considerable correlations between the results of surface electromyography and the results of HB and SB Grading Scales [24,25].

The HB scale provides a gross impression by classifying patients with IBP into 6 grades. The HB grading system involves making measurements of the movement of the eyebrow and corner of the mouth and comparing the results with those on the unaffected side. A scale with 0.25 cm divisions is used for the measurements; there is a total possible score of 8 (0.25 cm or 1 point, 1 cm or 4 points for the mouth and 0.25 cm or 1 point, 1 cm or 4 points for the eyebrow) and these results can easily be converted to the 6-point scale. Group I represents normal facial movement with no weakness or synkinesis. A patient placed in group II would have only slight asymmetry of facial movements with possible slight synkinesis. The patient in group III has an obvious asymmetry with obvious secondary defects but has some forehead movement. The presence of forehead movement indicates that there has not been total degeneration of the nerve. The patient in group IV has obvious asymmetry, no forehead movement, and weakness with possible disfiguring synkinesis or mass action. When there is only slight movement of the face, no forehead movement, and not enough facial function return to have secondary defects, the patient is in group V. The absence of any movement or tone places the patient in group VI [24,26]. Due to the convenience and simplicity of the HB scale, it is the most widely used facial nerve grading system [24].

SB scale is a regional weighted scale based on evaluation of different regions including resting symmetry, symmetry of voluntary movement and severity of synkinesis to form one single composite score from 0 to 100. Firstly, the physician assesses the symmetry of the eye, cheek (nasolabial fold) and mouth at rest. Choices under each item are provided to be assigned a value of 0–2, and the sum is assigned a weighted factor of 5. Secondly, the physician is asked to rate facial movements during five standard facial expressions on a scale of 1–5 (1—no movement, 2—slight movement, 3—mild excursion, 4—near normal movement, and 5—normal movement). The values are added together and multiplied by 4. In the third step, the physician is to grade the severity of synkinesis on a four-point scale (1—none, 2—mild, 3—moderate, and 4—severe) during the five expressions as in the second step. The sum of synkinesis score is given the weighted factor of 1. An overall score is the weighted sum of the three parts [25].

Based on the correlation between HB and SB grading scales values, and the results obtained using these two scales, the respondents were divided into 6 groups, and met the criteria from both scales (Table 1) [27].

Pain threshold value was determined using the digital pressure algometer. The digital pressure algometer is a standardized device for objectively quantifying a pressure pain threshold. The device is gun-shaped with a rubber flat tip having an area of 0.5 cm^2^, connected to a computer via a pressure transducer. In the course of the examination, the tip of the device is placed perpendicular to the trigger point, and the patient is required to press a button at the slightest feeling of pain, which will register the values via a USB unit, in real time [28]. The pain threshold was determined at 6 retroauricular points located in the RAR on the left and right side, and each point was tested 3 times, with the aim of minimizing the subjectivization of the method. In order to avoid, to the greatest degree possible, patients’ subjectivity, alterations in mimic muscles in certain facial expressions were monitored during examination with the Wong–Baker Faces Pain Rating Scale [29], minimizing the probability of error. The pain threshold at trigger points represents the arithmetic mean of the values obtained and expressed as kPa/cm^2^.

### 2.4. Statistical Analysis

The data analysis was conducted using SPSS^®^ Statistics 20.00 (Statistical Package for the Social Sciences) and R Core Team (2020) version 4.0.3. (The R Project for Statistical Computing). Descriptive and physiological data are expressed as mean ± SD. Statistical significance was determined at the alpha level of 0.05. Also, normal distribution of the variables was assessed using the Shapiro–Wilk test. To compare pain threshold among the six groups, we used one-way analysis of variance (ANOVA), followed by a post hoc analysis (Tukey’s Honestly Significant Difference). Pain threshold among patients regarding the affected side was assessed by the two-way ANOVA (factors: group—II, III, IV, V, VI; side—affected/healthy side). To show a statistically significant difference between the groups of patients with IBP (degree of damage) in terms of frequency of pain, we use Pearson’s Chi-squared test.

## 3. Results

The present study comprised 220 respondents, of whom 142 are patients affected by IBP (male-to-female ratio was 1.18, mean age 41 ± 21 years, left-to-right ratio was 0.95) and 78 expressed as HS grade (male-to-female ratio was 1.14, mean age 39 ± 19 years). No significant difference in the group of IBP patients has been noted between male/female or left/right side incidence of palsy.

The average pain threshold in the right RAR in the group of HS is 462.25 ± 84.87 kPa/cm^2^, while in the left RAR is slightly higher and amounts to 466.66 ± 91.53 kPa/cm^2^. There is no statistically significant difference in the degree of the pain threshold between the right and left RAR. The average pain threshold in both sides in HS is 464.45 ± 85.50 kPa/cm^2^.

**Figure 1:**
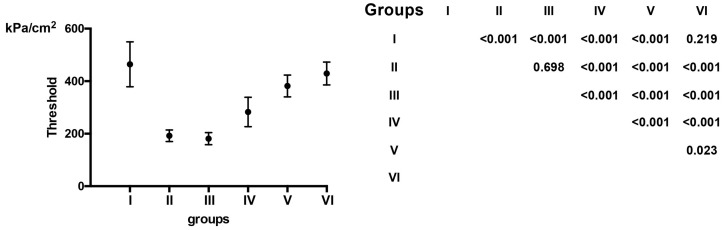
Pain threshold values in the RAR of healthy controls and patients with Bell’s palsy on the affected side. Pain threshold levels of each group are presented as average (lack dots) ± standard deviation (whiskers). One-way ANOVA revealed a statistically significant difference among the groups and Tukey’s HSD post hoc *p* values are presented.

Pain threshold values in patients with IBP on the affected side were 192.01 ± 22 kPa/cm^2^ in group II, 181.34 ± 23.32 kPa/cm^2^ in group III_,_ 282.93 ± 55.98 kPa/cm^2^ in group IV, 382.00 ± 41.57 kPa/cm^2^ in group V, whereas it was 429.44 ± 43.73 kPa/cm^2^ in group VI. Pain threshold values in the RAR-affected side in the group of HS and patients with IBP, and post hoc *p* values among the groups revealed by the one–way ANOVA are presented in Figure 1.

**Figure 2:**
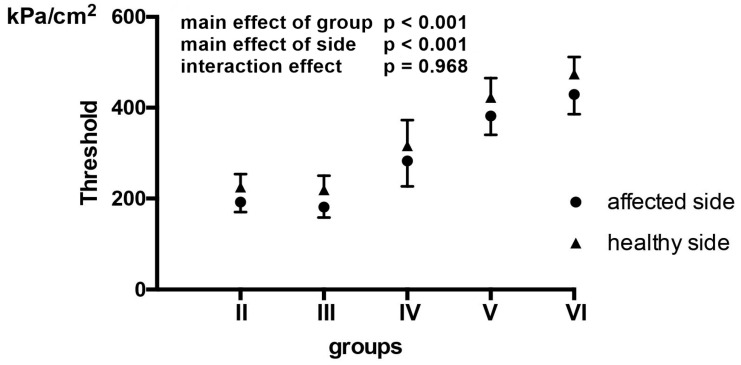
Pain threshold values among patients with Bell’s palsy regarding the affected side. Pain threshold levels of the affected side (black dots) and the healthy side (black triangles) are presented as averages ± standard deviation (whiskers). Pain threshold was assessed by two-way ANOVA (factors: group—II, III, IV, V, VI; side—affected/healthy side). There was no interaction effect (*p* = 0.968), but a significant effect of group (*p* < 0.001) and side (*p* < 0.001).

Pain threshold values in patients with IBP on the healthy side in II group are 225.21 ± 28.43 kPa/cm^2^, in III group is 219.27 ± 31.23 kPa/cm^2^, in IV group is 316.44 ± 76.63 kPa/cm^2^, in V group is 423.00 ± 18.94 kPa/cm^2^ while in VI group is 474.02 ± 36.28 kPa/cm^2^. Pain threshold values in patients with IBP in the RAR-affected side and healthy side, as well as *p*-values among the groups revealed by two-way ANOVA are presented in Figure 2.

In our series of patients with IBP, RAP was present in 57.7% of the whole study group. RAP preceded the onset of the palsy in 26.8% of cases, while after the onset of palsy it occurred in 17.6% of cases. The presence of RAP in examined groups of patients with IBP at different stages of IBP are presented in Table 2.

Using Pearson’s Chi-squared test, we showed that there is a statistically significant difference between the groups of patients (degree of damage) in terms of frequency of pain (X-squared = 39.093, *p*-value < 0.001).

The incidence (frequencies) of RAP that precedes paralysis and ceases after its occurrence in groups II and III of patients with IBP (0% and 8.6%) is noticeably lower than expected frequencies (as calculated by default in Chi-squared test for contingency tables) and that the incidence of RAP that occurred only after the onset of paralysis (46.2% and 31.4%) is noticeably more frequent than the expected frequency.

The results also showed that the incidence of RAP that precedes paralysis and ceases after its occurrence in groups V and VI of patients with IBP (42.4% and 64.3%) is more frequent than expected. Although the incidence of RAP that occurred only after the onset of paralysis (6.1% and 0%) is not statistically noticeably less frequently than expected, there is a trend which suggested that if we increase the number of the respondents the incidence will be statistically noticeably.

In group IV patients with IBP, we did not prove a statistically significantly lower or more frequent frequency of RAP than expected.

In the groups of IBP patients in which pain was presented before and after the onset of the paralysis as well as in the groups of IBP patients in which the pain was never presented, we did not prove a statistically significantly lower or more frequent frequency than expected.

## 4. Discussion

Jong and colleagues demonstrated that patients with a higher degree of IBP have a higher degree of FN damage [24,30].

RAP in patients with IBP has also been described by other studies [12,15,19]. Findings of this study are in accordance with the results reported by the other authors [12,15]. The pathogenesis of RAP in IBP is not entirely clear. The anoxia of the FN, caused by primary or secondary ischemia, followed by compensatory dilatation of the blood vessels supplying the FN, is part of the process causing the occurrence of RAP, and RAP would then result from the dilation phase of the vessels. The intermediate nerve has a branch carried via the greater petrosal nerve that carries sensations from the skin in the region of the external ear and mastoid region. Inflammation of FN involving this nerve branch would result in an ipsilateral pain in this area [1,18]. The role of nervi nervorum (NN) in RAP in IBP has been explained by Han [19]. The FN, alike all peripheral nerves, has free nerve endings in the perineurium and endoneurium, derived from fibers in the nerve trunk itself, which have nociceptive function. When a peripheral nerve is damaged, three types of pain can be produced. First, nerve trunk pain is attributed to increased activity in mechanically or chemically sensitized nociceptors within the nerve sheath. Second, dysesthetic pain, which is attributed to damaged nociceptive afferent axons themselves, and third, there can be referred pain from the nerve sheaths innervated by NN through central convergence. Thus, the facial pain and pain at nerve exit through the stylomastoid foramen would be explained by the involvement of the nerve trunk nociceptors, while the RAP would be primarily generated by algogenic stimuli from the sheaths of FN delivered to trigeminocervical nuclear complex via NN [1,19].

FN is richly innervated by their nerves, NN, that have nociceptive function. Stimulation of NN dominating in FN trunk can be transmitted to trigeminocervical nuclear complex and make referred pain in the craniofacial region segmentally. The reason why referred pain of FN origin develops around the ear is that FN and its unsheathed connective tissue are derivatives of the second branchial arch, which is homologous with the somites of the body [19].

The fibers of these tiny nerves belong to group A Delta [31,32], which is most sensitive to pressure [33]. In mild and moderate degrees of FN trunk damage (neuropraxia), the injury may result in some form of localized damage to the myelin sheath. There is no axonal degeneration so that the axonoplasmic continuity remains intact distal to the lesion. FN, in addition to the resulting neuropraxic injuries, will continue to conduct neural impulses while stimulation of NN can be transmitted to the trigeminocervical nuclear complex and make referred pain in the RAR and hyperalgesia along the second branchial arch including the dermis, subcutaneous tissue, and muscles around the ear. It could have the potential to evoke tenderness of these muscles and use it as a warning sign of IBP, coexistent with RAP. Thus, it would be worthwhile to use it as a warning sign because referred pain with lower pain threshold and tenderness appear [1,19,34,35].

However, in the more severe cases of IBP, neurotmesis can occur besides neuropraxia and axonotmesis. Consequently, it is manifested by the micro-necrosis of NN, the termination of conduction of afferent impulses, and the absence of RAP and eventually causing the more severe FN damage. After being damaged, fibers will not transmit efferent impulses and innervate mimic muscles, but neither will they transmit afferent impulses leading to RAP. There is a disproportion between the current pain sensation and the degree of muscle loss, whereas the pain that occurs before the onset of facial paralysis indicates not only the latent period of NN injury but also the onset of more severe forms of IBP [35,36,37].

Based on the findings of this study, we suggest that the occurrence of retroauricular pain before the onset of facial weakness is associated with a higher severity of IBP, while the occurrence after the onset of weakness is associated with lower severity of IBP. We can explain these results by the fact that the FN fibers and NN in patients belonging to groups II, III, IV, and V are still able to convey impulses in both afferent and efferent directions, and therefore the occurrence of RAP after the onset of the paralysis cannot reliably ascertain the more severe degree of FN damage [1,15,16,17]. The prodromal sign onset in IBP and FN paresis begins a few days before the onset of mimic musculature paralysis [12], which is associated with the cause of RAP at this time point. FN is damaged and therefore stimulates the nociceptor, but the nerve fiber is still able to transmit impulses smoothly in both efferent and afferent direction, respectively [35,37].

Patients belonging to groups II, III, IV, and V also had a statistically significantly lower pain threshold in the RAR side, opposite to the paresis, compared to HS and patients belonging to group VI. This phenomenon, when the patients sense pain in the contralateral side, is known as mirror-image pain. Pathogenesis of mirror-image pain is still not exactly known, but there are some possible signaling mechanisms in the contralateral reaction of the nerve tissue after unilateral nerve injury [38,39,40]. If the RAP were observed exclusively from a nociceptive point of view, central sensitization would increase the transmission of peripheral stimuli and cause secondary hyperalgesia [19,37]. It would be expected that the pain threshold in the RAR side, opposite to paralysis, is lower in patients with more severe impairment. On the other hand, since RAP is primarily referred to the pain (of transmitted—projected origin), NN play the most important role in the conduction of afferent impulses. Thus, pathologically affected NN is associated with the impaired impulse conduction within the peripheral afferents, resulting in the absence of central sensitization in cases of severe degree of damage. Accordingly, in the cases of a mild form of nerve impairment, responsiveness of nociceptive neurons in the central nervous system is increased due to stimuli transmitted through the NN on the affected FN side, whereas on the opposite side, pain threshold decreases as a possible effect of ephaptic coupling, electrical interactions between functionally independent adjacent unmyelinated axons on the affected and opposite healthy-unaffected side. Based on the obtained results that patients with the more severe damage have shown a higher pain threshold on the side opposite to the injury, we can conclude that the nociceptive component of pain represents a smaller percentage of the origin of pain correlated with a severe grade of IBP due to nociceptive deafferentation, thus preventing ephaptic transmission [19,41,42,43,44,45,46].

In this study, we obtained results that there is pain (referred pain), a lowered pain threshold on the damaged side and on the opposite, motor healthy side. In this conversation, we could call it a “mirror image lowered pain threshold”, or simply “mirror lowered pain threshold” [38,39,40].

## 5. Conclusions

In this study, we found no difference in the degree of the pain threshold between the right and left RAR in the group of HS. Patients with Bell’s palsy belonging to groups II, III, IV, and V had lower pain thresholds in both RARs than HS. We also found no difference in the degree of the pain threshold in both RARs between HS and patients belonging to group VI. The degree of pain threshold lowering in RAR (bilaterally) is inversely related to the severity of IBP. There was also no difference in the degree of pain threshold in RAR between the affected and unaffected side in the group of patients with IBP. The incidence of RAP that precedes paralysis and ceases after its occurrence in groups II and III of patients with IBP is noticeably lower and the incidence of RAP that occurred only after the onset of paralysis is more frequent in our study. The results also showed that the incidence of RAP that precedes paralysis and ceases after its occurrence in groups V and VI of patients with IBP was more frequent. We suggest that the occurrence of retroauricular pain before the onset of facial weakness is associated with a higher severity of IBP, while the occurrence after the onset of weakness is associated with lower severity of IBP.

## Figures and Tables

**Table 1:** Groups of patients with idiopathic Bell’s palsy (IBP) and comparison between House-Brackman (HB) and SunnyBrook (Sb) grading scale values [27].

Group of IBP	House–Brackmann	Sunnybrook
Group I	I	100
Group II	II	70–99
Group III	III	43–69
Group IV	IV	26–42
Group V	V	13–25
Group VI	VI	0–12

Mervi Kanerva, Lars Jonsson, Thomas Berg et al. Department of Otorhinolaryngology, University Central Hospital, Helsinki, Finland. Otolaryngology—Head and Neck Surgery; 144 (4) p. 574, copyright © 2011 by SAGE Publications. Reprinted (adopted) by Permission of SAGE Publications, Inc (Thousand Oaks, California, USA).

**Table 2:** The presence of RAP (retroauricular pain) in different groups of patients at different stages of IBP.

Group	Pain Presented Just Before the Onset of Paralysis	Pain Presented Just After the Onset of Paralysis	Pain Presented Before and After the Onset of Paralysis	Pain Never Presented
II	0/13 (0%)	6/13 (46.2%)	0/13 (0%)	7/13 (53.8%)
III	3/35 (8.6%)	11/35 (31.4%)	6/35 (17.1%)	15/35 (42.9%)
IV	12/47 (25.5%)	6/47 (12.8%)	5/47 (10.6%)	24/47 (51.1%)
V	14/33 (42.4%)	2/33 (6.1%)	5/33 (15.2%)	12/33 (36.4%)
VI	9/14 (64.3%)	0/14 (0%)	2/14 (14.3%)	3/14 (21.4%)
